# Testosterone Supplementation in Patients With Chronic Heart Failure: A Meta-Analysis of Randomized Controlled Trials

**DOI:** 10.3389/fendo.2020.00110

**Published:** 2020-03-13

**Authors:** Jianping Tao, Xiaoyong Liu, Wenwei Bai

**Affiliations:** ^1^Department of Anesthesiology, The Second Affiliated Hospital of Kunming Medical University, Kunming, China; ^2^Department of Cardiology, The Second Affiliated Hospital of Kunming Medical University, Kunming, China

**Keywords:** testosterone, heart failure, exercise capacity, randomized controlled trials, meta-analysis

## Abstract

**Background:** The effect of testosterone supplementation in patients with chronic heart failure (CHF) remains uncertain.

**Methods:** A meta-analysis of randomized controlled trials (RCTs) was performed. RCTs that evaluate the chronic effect of testosterone supplementation on exercise capacity and cardiac function in CHF were identified via searching of PubMed, Embase, and the Cochrane's Library databases. Heterogeneity was evaluated by the Cochrane's Q test and *I*^2^ statistics. A fixed-effect model was used if the heterogeneity was not significant (*I*^2^ < 50%); otherwise, a random-effect model was applied.

**Results:** Eight studies including 170 patients in the testosterone supplementation group and 162 in the control group were included. Overall, testosterone supplementation was not associated with an improved exercise capacity (walking test: standardized mean difference [SMD] = 0.36, *p* = 0.07). Sensitivity analyses limited to male patients showed similar results (SMD = 0.21, *p* = 0.15), and subgroup analyses also showed similar results in male HF patients with baseline total testosterone (TT) ≥ or < 10 nmol/L. However, patients with TT at endpoint ≥ 25 nmol/L was associated with improved exercise capacity (SMD = 1.12, *p* = 0.02), but not for those with TT at endpoint < 25 nmol/L (SMD = 0.24, *p* = 0.12). In addition, VO_2max_ (weight mean difference [WMD] = 0.85, *p* = 0.43), the functional classification (the New York Heart Association classification: WMD = −0.08, *p* = 0.16) and quality of life (Minnesota Living with Heart Failure [MLHF] questionnaire: WMD = −6.03, *p* = 0.12) were not significantly affected. Moreover, testosterone supplementation did not significantly affect left ventricular ejection fraction (WMD: −1.52%, *p* = 0.37), serum B-type natriuretic peptide (SMD: −0.19, *p* = 0.23), or a composite outcome of death or HF hospitalization (risk ratio [RR]: 1.02, *p* = 0.96). Although testosterone supplementation increased systolic blood pressure (BP) in CHF patients (WMD: 5.68 mmHg, *p* < 0.001), diastolic BP or heart rate was not significantly changed as compared to control.

**Conclusions:** Testosterone supplementation within a physiological range is not associated with significantly improved exercise capacity, cardiac function, quality of life, or clinical outcome in CHF patients.

## Introduction

Chronic heart failure (CHF) is a severe clinical syndrome that occurs as a late stage of various cardiovascular diseases (1). Pathophysiologically, CHF is characterized by cardiac dysfunction and neurohormonal activation, such as the sympathetic nervous system, renin-angiotensin-aldosterone system (RAAS), inflammatory system, and oxidative stress system etc. (2). Although evidenced-based medical therapy for CHF have been improved in recent decades (3–5), CHF remains one of leading causes of morbidity and mortality worldwide (6, 7). Therefore, identification of potential treatment target for CHF remains of great clinical importance.

Imbalance of anabolic/catabolic balance has been recognized as an important feature in patients with CHF, which may be related to the deficiency of testosterone, an anabolic hormone of the body (8, 9). Testosterone deficiency has been related with the severity of cardiac dysfunction and poor prognosis in CHF patients (10, 11). Accordingly, randomized controlled trials (RCTs) have been performed to evaluate the efficacy of testosterone supplementation in CHF patients (11–13). However, the results of the RCTs were not always consistent (14–21). Moreover, the sample sizes of the RCTs were generally small (20–70 participants in each RCT), which may limit the statistical power of these studies. Although a previously published meta-analysis have systematically evaluated the efficacy of testosterone supplementation in CHF patients (22), only four RCTs (14–17) were included and many outcomes such as the influences of testosterone supplementation on cardiac functional classification, quality of life, blood pressure (BP) and resting heart rate (HR), were not quantitatively evaluated since limited RCTs were available. A few RCTs (18–21) have been published since the last meta-analysis. Therefore, in this study we aimed to perform an updated meta-analysis to systematically evaluate the efficacy of testosterone supplementation in patients with CHF.

## Methods

This systematic review and meta-analysis was performed in accordance to the PRISMA (23) (Preferred Reporting Items for Systematic Reviews and Meta-Analyses) statement and the Cochrane Handbook (24) guidelines.

### Search Strategy

PubMed, Embase, and the Cochrane Library (Cochrane Center Register of Controlled Trials) were systematically searched for relevant RCTs, using the combination of the following three groups of terms: (1) “testosterone,” “androgen,” “dihydrotestosterone,” or “DHT”; (2) “heart,” “cardiac,” or “ventricular,” couple with “failure,” “insufficiency,” “dysfunction,” or “inadequacy,” or “cardiomyopathy”; and (3) “random,” “randomized,” “randomly,” or “randomization.” The search was limited to studies in humans, and we did not apply restriction to the type of HF (acute or chronic) during the literature search stage as to avoid missing of relevant studies. We also analyzed reference lists of the original and review articles using a manual approach. The final searching was performed on August 15th, 2019.

### Study Selection

Studies were included if they met the following criteria: (1) full-length articles published in English; (2) reported as RCTs with a parallel design; (3) included patients with stable CHF (with no restrictions to the gender of the patients, etiologies of CHF, or the systolic function) who received evidence-based medications for CHF; (4) patients were randomly assigned to a treatment group of testosterone (with no restrictions to the dose or the administrative routes of the supplementation), and a control group with placebo or no treatment; (5) with a follow-up duration of at least one month because we did not want to observe the acute effect of testosterone supplementation; and (6) reported at least one of the following outcomes, primary outcome: exercise capacity as indicated by the results of the shuttle walk test (SWT) or the six-minute walk test (6MWT); and secondary outcomes, including functional capacity of maximal oxygen consumption (VO_2max_) in cardiopulmonary exercise test, changes of the New York Heart Association Class, quality of life as indicated by the result of Minnesota Living with Heart Failure (MLHF) questionnaire; parameters reflecting cardiac function, such as left ventricular ejection fraction (LVEF), brain natriuretic peptide (BNP) or N-terminal pro brain natriuretic peptide (NT-proBNP); hemodynamic parameters, including systolic and diastolic blood pressure (SBP and DBP), as well as resting heart rate (HR). Moreover, incidence of the composite outcome of all-cause mortality or HF rehospitalization, as well as adverse events that were deemed to be associated with testosterone supplementation were also investigated. Reviews, observational studies, crossover studies, and studies in which the outcomes of interest were not reported or unavailable were excluded from the current study.

### Data Extraction and Quality Assessment

Two authors independently performed the literature search, data extraction, and quality assessment according to inclusion criteria. Discrepancies were resolved by consensus. Extracted data included the design characteristics, baseline characteristics of the included patients (age, gender, comorbidities, baseline cardiac systolic function as reflected by LVEF and BNP, etiologies of CHF, and the proportions of patients with heart failure with reduced ejection fraction [HFrEF], and concurrent medications), regimens of testosterone supplementation (doses and administrative routes), mean serum levels of total testosterone (TT) at baseline and endpoint in patients allocated to testosterone supplementation and control groups, as well as follow-up durations. We used the seven domains of the Cochrane Risk of Bias Tool (24) to evaluate the quality of the included studies, which include criteria concerning sequence generation, allocation concealment, blinding of participants and personnel, blinding of outcome assessors, incomplete outcome data, selective outcome reporting and other potential threats to validity.

### Statistical Analysis

Continuous variable was analyzed using weighted mean difference (WMD), whereas dichotomous data was analyzed using risk ratios (RR) with 95% confidence interval (CI). Standardized mean difference (SMD) was used for continuous variable if an outcome was analyzed by different ways (such as exercise capacity was measured via SWT and 6MWT), as recommended by the Cochrane's Handbook (24). Cochrane's Q test was applied to evaluate the heterogeneity among the included studies, and significant heterogeneity was considered for *P* < 0.10. Also determined was the *I*^2^ statistic, which describes the percentage of total variation across studies that is due to heterogeneity rather than chance (25, 26). An *I*^2^ > 50% indicated significant heterogeneity among the trials. A fixed-effect model was used to pool the results if the heterogeneity was not significant (*I*^2^ <50%); otherwise, a random-effect model was applied (26). Predefined sensitivity and subgroup analyses (24) were used to evaluate whether the efficacies of testosterone supplementation were consistent in men or women. Moreover, for the primary outcome of exercise capacity, to evaluate the potential influences of gonad status on the outcome, subgroup analyses were also performed according to the mean serum TT levels at baseline and at the study endpoint in male HF patients that received testosterone supplementation. The median of the mean TT levels among the included studies were chosen as the cutoff value for stratification. Potential publication bias was assessed with Egger's regression asymmetry test, or visual inspection of funnel plots if limited RCTs are included (27). P values were two-tailed and statistical significance was set at 0.05. We used RevMan software for the meta-analysis and statistical study (Version 5.1; Cochrane, Oxford, UK) and Stata software (Version 12.0; Stata, College Station, TX).

## Results

### Search Results

**Figure 1:**
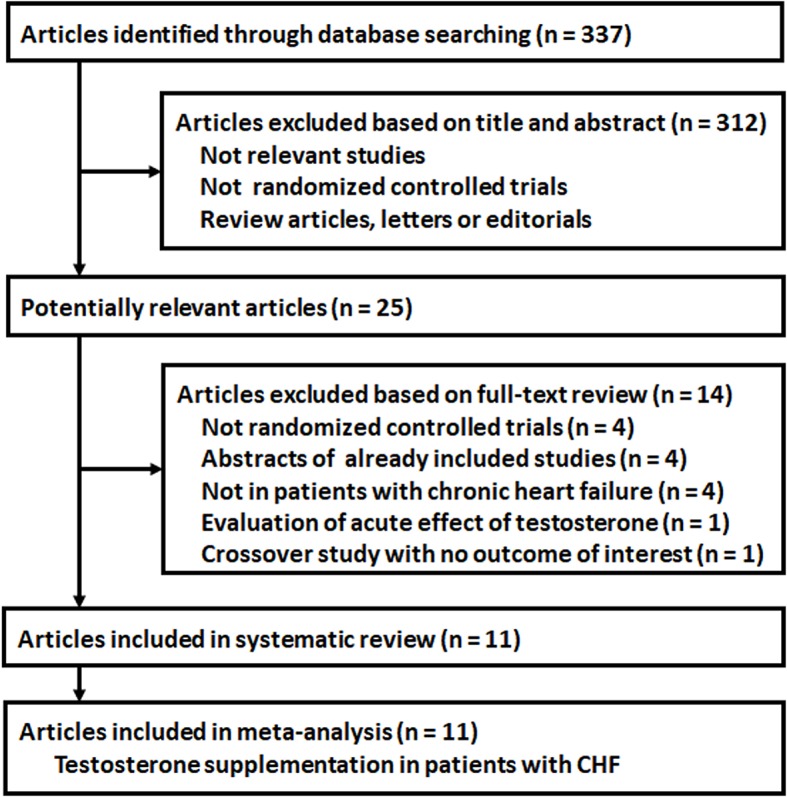
Flowchart of database search and literature identification.

A total of 337 articles were identified through database search, and 312 were excluded based on title and abstract screening, mainly because these studies were not relevant to the aim of the meta-analysis. Of the 25 potentially relevant articles, eleven (14–21, 28–30) met the inclusion criteria for the current meta-analysis (Figure 1). Fourteen articles were further excluded because four of them were not RCTs, four were abstracts of already included studies, four were not in patients with CHF, one evaluated the acute effect of testosterone, and the other one was a crossover study that did not report related outcome. For the eleven included articles, eight (14–21) were publications of the main findings of the eight RCTs, while the other three (28–30) were the reports of the additional outcomes of already included RCTs. These articles were all included for systematic review and meta-analysis.

### Study Characteristics

Overall, eight RCTs with a total of 332 CHF patients from eleven articles (14–21, 28–30) were included, including 170 patients in the testosterone supplementation group and 162 in the control group. The characteristics of the included studies were listed in Tables 1, 2. Briefly, all of the included RCTs were double-blinded (14–21), and seven studies were placebo-controlled studies except one study which applied blank treatment in control group (20). Six of the RCTs were performed in Europe (14–18, 21), one in South America (20), and another one in Asia (19). The mean ages of the included patients were between 50 and 70 years. Seven (14–16, 18–21) of the RCTs included men only, while the other one (17) included female patients (Table 1). Myocardial ischemia was the cause of CHF for more than half of the patients, and all of them were with reduced ejection fraction. All of the included patients with CHF received optimal medical treatments (Table 2). Different doses and administrative routs of testosterone (intramuscularly [i.m.] or via a skin patch) were used. In six studies (14, 16, 18–21), i.m. testosterone was applied with doses of 100–100 mg per 2 weeks to 3 months (Table 2). In the other two studies (15, 17), skin patches of testosterone were applied with 5 mg every 24 h or 300 μg 2 times a week (Table 2). The follow-up durations were between three to 12 months. The TT level for the treated group at baseline and endpoint were 1.4 and 3.5 nmol/L in the study including female patients only (17), and were 7.6–15.8 and 17.2–36.6 nmol/L in studies including male CHF patients (14–16, 18–21).

**Table 1:** Patient characteristic of the included studies.

**Study**	**Country**	**Design**	**No. of patients**	**Age**	**Male**	**BMI**	**Ischemic etiology**	**HFrEF**	**NYHA class**	**LVEF**	**T2DM**	**AF**	**SBP**	**DBP**
				**Years**	**%**	**kg/m2**	**%**	**%**		**%**	**%**	**%**	**mmHg**	**mmHg**
Pugh et al. (14)	UK	R, DB, PC	20	61.5	100	28.8	60.0	100	2.4	35.0	NR	NR	133	78
Malkin et al. (15)	UK	R, DB, PC	76	64.0	100	27.8	53.9	100	2.5	33.5	17.1	NR	130	78
Caminiti et al. (16)	Italy	R, DB, PC	70	70.0	100	26.3	77.1	100	2.5	32.7	28.6	37.1	127	91
Iellamo et al. (17)	Italy	R, DB, PC	32	68.6	0	27.9	100.	100	3.0	32.9	53.1	28.1	114	78
Stout et al. (18)	UK	R, DB, PC	28	67.2	100	29.3	71.4	100	2.5	24.4	32.1	35.7	138	82
Mirdamadi et al. (19)	Iran	R, DB, PC	50	60.5	100	26.5	NR	100	2.4	32.1	26.0	NR	123	80
Dos Santos et al. (20)	Brazil	R, DB	27	52.0	100	NR	51.8	100	3.0	25.5	NR	NR	115	67
Navarro-Penalver et al. (21)	Spain	R, DB, PC	29	65	100	30	62	100	2.3	30	48.3	37.9	NR	NR

*BMI, body mass index, HFrEF, heart failure with reduced ejection fraction; NYHA, New York Heart Association; LVEF, left ventricular ejection fraction; T2DM, type 2 diabetes mellitus; AF, atrial fibrillation; SBP, systolic blood pressure; DBP, diastolic blood pressure; NR, not reported; R, randomized; DB, double blinded; PC, placebo controlled*.

**Table 2:** Treatment characteristics of included studies.

**Study**	**β-blockers**	**ACEIs/ARBs**	**ARAs**	**Diuretics**	**Digoxin**	**Anti-platelets**	**Statins**	**Testosterone regimen**	**Control**	**TT at baseline**	**TT at end point**	**Follow-up duration**
	**%**	**%**	**%**	**%**	**%**	**%**	**%**			**nmol/L**	**nmol/L**	**months**
Pugh et al. (14)	35	100	20	80	40	95	90	100 mg i.m. every 2 weeks	*P*	T: 15.8 C: 13.9	T: 36.6 C: 12.2	3
Malkin et al. (15)	44.7	100	NR	73.7	46.1	NR	NR	5 mg skin patch every 24 h	*P*	T: 13.9 C: 12.1	T: 19.5 C: 12.6	12
Caminiti et al. (16)	87.1	92	41	47.1	33	61.4	78.6	1,000 mg i.m. every 6 weeks	P	T: 8.0 C: 7.3	T: 18.0 C: 8.0	3
Iellamo et al. (17)	81.3	91	60	78	32	100	78.1	300 μg transdermal patch 2 times a week	*P*	T: 1.4 C: 1.4	T: 3.5 C: 1.0	6
Stout et al. (18)	78.6	92.9	14.3	46.4	7.1	57.1	78.6	100 mg i.m. every 2 weeks	*P*	T: 10.4 C: 11.2	NR	3
Mirdamadi et al. (19)	NR	NR	NR	NR	NR	NR	NR	250 mg i.m. every 4 weeks	*P*	NR	NR	3
Dos Santos et al. (20)	100	77.8	63	77.8	40.7	NR	NR	1,000 mg i.m. 1–2 times	No treatment	T: 7.7 C: 8.6	T: 17.2 C: 14.9	4
Navarro-Penalver et al. (21)	96.6	100	86.2	100	27.6	NR	NR	1,000 mg im q3m	*P*	T: 7.6 C: 8.9	T: 18.4 C: 10.9	12

*ACEIs, angiotensin converting enzyme inhibitors; ARBs, angiotensin II receptor blockers; ARAs, aldosterone receptor antagonist; NR, not reported; i.m., intramuscularly; TT, total testosterone; T, treatment group with testosterone; C, control group*.

### Data Quality

**Figure 2:**
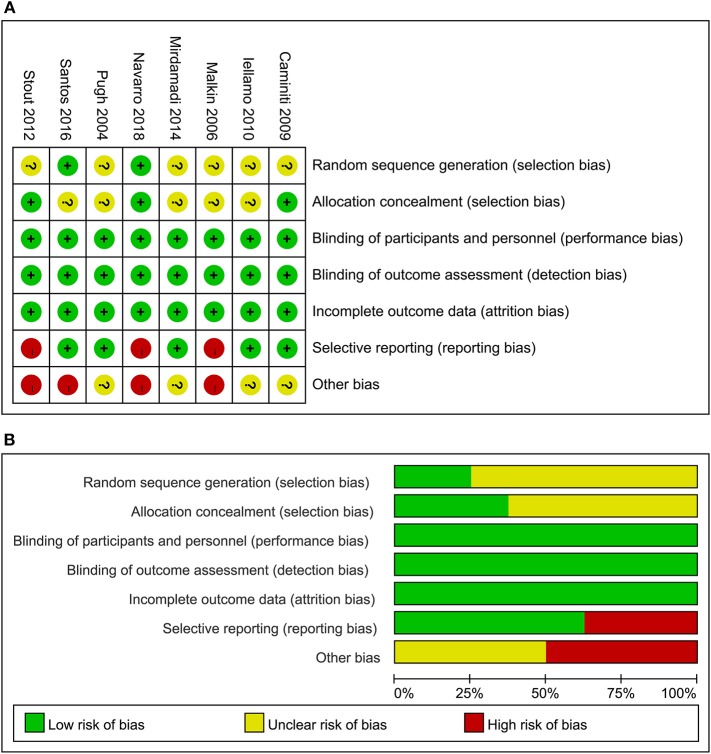
Summary of study quality evaluation according to the Cochrane's Risk of Bias Tool; **(A)** details of quality evaluation for each of the included RCTs according to the seven domains of the Cochrane's Risk of Bias Tool; and **(B)** summary of risk of bias of the RCTs included in the meta-analysis.

The details of risks of biases of the included studies according to the Cochrane assessment tool are listed in Figure 2. Details of random sequence generation were reported in two studies (20, 21), and the strategies of allocation concealment were reported in three studies (16, 18, 21). Details of withdrawals and dropouts were reported in all studies.

### Effects of Testosterone on Exercise Capacity

**Figure 3:**
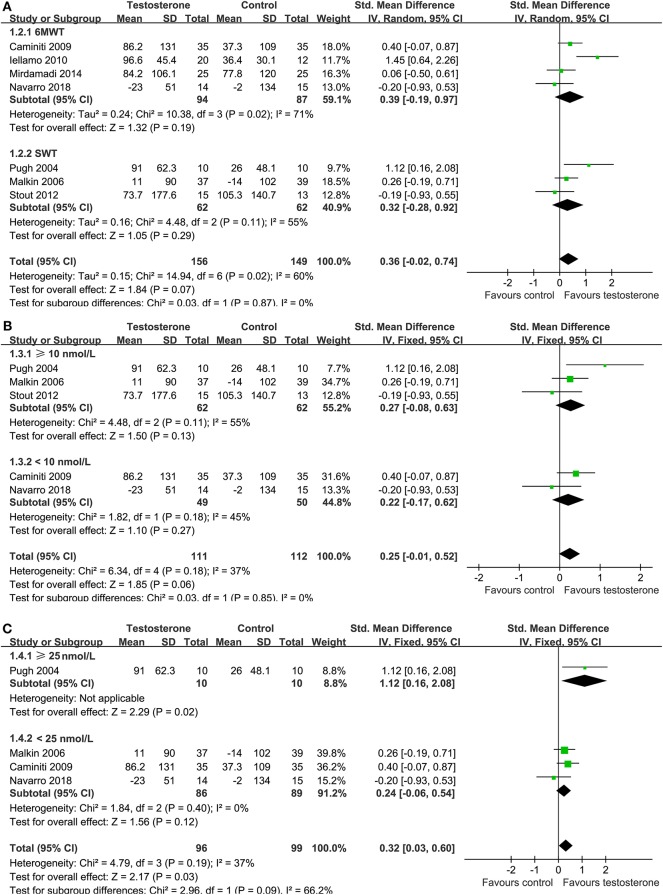
Forest plots for the meta-analysis of the effect of testosterone supplementation on exercise tolerance; **(A)** exercise capacity as evaluated by the shuttle walk test (SWT) or the six-minute walk test (6MWT); **(B)** subgroup analysis according to the baseline mean total testosterone level in male CHF patients treated with testosterone supplementation; and **(C)** subgroup analysis according to the mean total testosterone level at endpoint in male CHF patients treated with testosterone supplementation.

Overall, meta-analysis with seven studies (14–19, 21), four with the 6MWT (16, 17, 19, 21) and the other three with SWT (14, 15, 18), showed that testosterone supplementation was not associated with a significantly improved exercise capacity as compared with control (random-effect model; SMD = 0.36, 95% CI: −0.02 to 0.74, *p* = 0.07; Figure 3A) with significant heterogeneity (*I*^2^ = 60%). Subgroup stratified by the test showed that testosterone supplementation did not significantly affect the exercise capacity as reflected by the performance in 6MWT (SMD = 0.39, *p* = 0.19) or in SWT (SMD = 0.32, *p* = 0.29). Sensitivity analysis by omitting the study with women only did not significantly affect the result (SMD = 0.21, 95% CI: −0.08 to 0.50, *p* = 0.15). Subgroup analyses showed that testosterone supplementation did not significantly affect the exercise capacity in male CHF patients with baseline TT ≥ 10 nmol/L (three studies, SMD = 0.27, 95% CI: −0.08 to 0.06, *p* = 0.13) or those with baseline TT <10 nmol/L (two studies, SMD = 0.22, 95% CI: −0.17 to 0.62, *p* = 0.27; Figure 3B). However, subgroup analyses according to the TT level at endpoint showed that male patients with endpoint TT ≥ 25 nmol/L was associated with improved exercise capacity (one study, SMD = 1.12, 95% CI: 0.16 to 2.08, *p* = 0.02), but not for those with endpoint TT <25 nmol/L (SMD = 0.24, 95% CI: −0.06 to 0.54, *p* = 0.12; Figure 3C).

### Effects of Testosterone on NYHA Function and MLHF Scores

**Figure 4:**
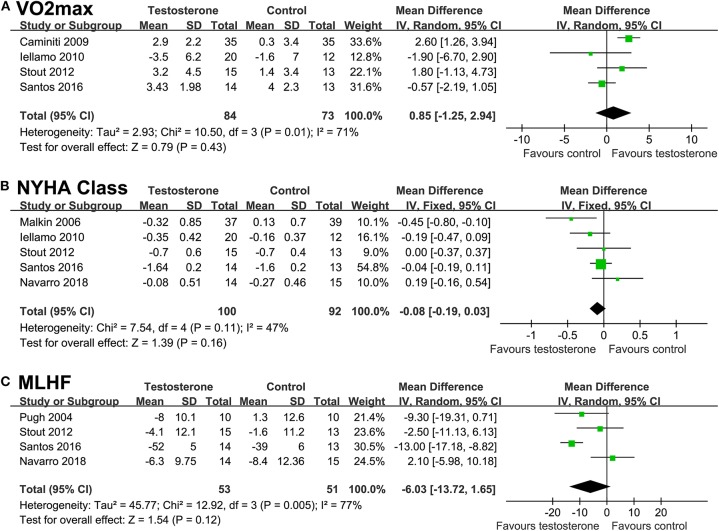
Forest plots for the meta-analysis of the effect of testosterone supplementation on functional capacity, cardiac functional classification, and quality of life in CHF patients. **(A)** functional capacity of maximal oxygen consumption (VO_2max_) in cardiopulmonary exercise test; **(B)** New York Heart Association (NYHA) functional classification; and **(C)** the quality of life as indicated by the result of Minnesota Living with Heart Failure (MLHF) questionnaire.

Testosterone supplementation was not associated with a significant improved VO_2max_ in cardiopulmonary test (random-effect model; WMD = 0.85 ml/kg/min, 95% CI: −1.25 to 2.94, *p* = 0.43; Figure 4A), with similar result in sensitivity analysis limited to studies of male CHF patients only (WMD = 1.25 ml/kg/min, 95% CI: −0.99 to 3.49, *p* = 0.27). Similarly, neither NYHA classification (WMD = −0.08, *p* = 0.16; Figure 4B) nor MLHF score (WMD = −6.03, *p* = 0.12; Figure 4C) were significantly improved after testosterone supplementation.

### Effects of Testosterone on Cardiac Function, Clinical Outcome, Blood Pressure, and Resting HR

**Figure 5:**
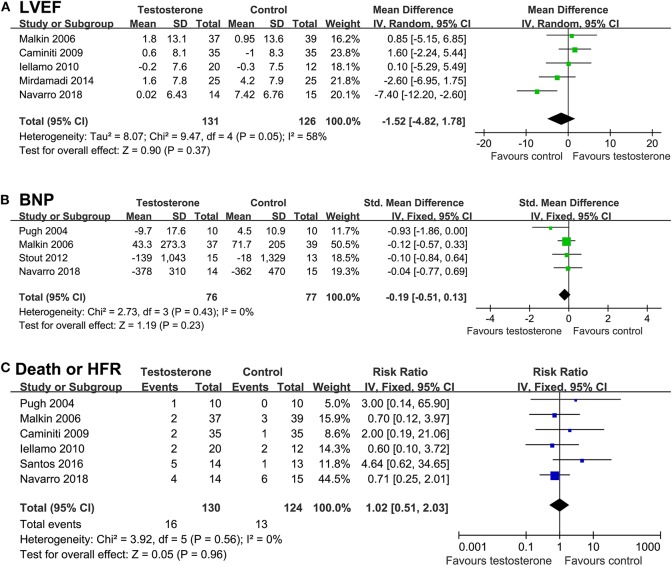
Forest plots for the meta-analysis of the effect of testosterone supplementation on cardiac function and clinical outcomes in CHF patients. **(A)** left ventricular ejection fraction (LVEF); **(B)** brain natriuretic peptide (BNP); and **(C)** a composite outcome death or HF rehospitalization.

**Figure 6:**
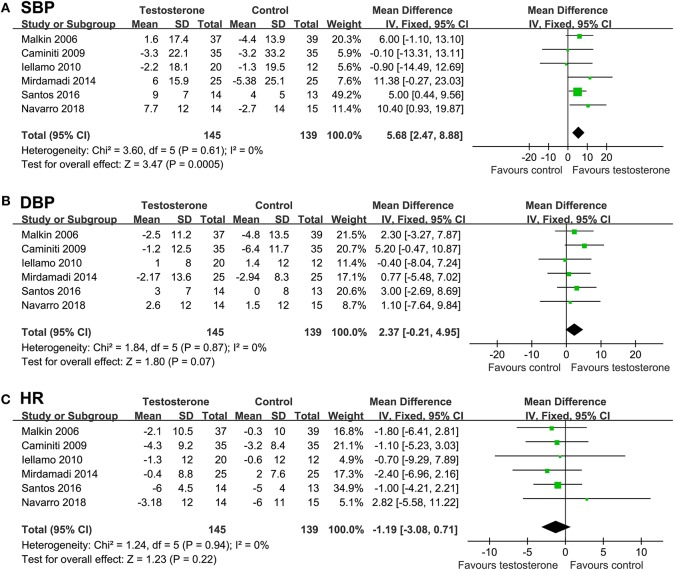
Forest plots for the meta-analysis of the effect of testosterone supplementation on hemodynamic parameters in CHF patients. **(A)** systolic blood pressure (SBP); **(B)** diastolic blood pressure (DBP); and **(C)** resting heart rate (HR).

Result of meta-analyses showed that testosterone supplementation was not associated with significant improved LVEF (WMD = −1.52%, *p* = 0.37; Figure 5A) or BNP (SMD = −0.19, *p* = 0.23; Figure 5B) as compared with controls. Moreover, meta-analyses with six studies (14–17, 20, 21) showed that testosterone supplementation did not significantly affect the risk of the composite outcome of death or HF rehospitalization (RR = 1.02, 95% CI: 0.51 to 2.03, *p* = 0.96; *I*^2^ = 0%; Figure 5C), and the meta-analysis limited to male CHF patients showed similar results (RR = 1.11, *p* = 0.78). Pooled results showed that although testosterone supplementation significantly increased SBP in CHF patients (WMD: 5.68 mmHg, *p* < 0.001; Figure 6A), DBP or HR were not significantly changed compared to control group (Figures 6B,C).

### Publication Bias

**Figure 7:**
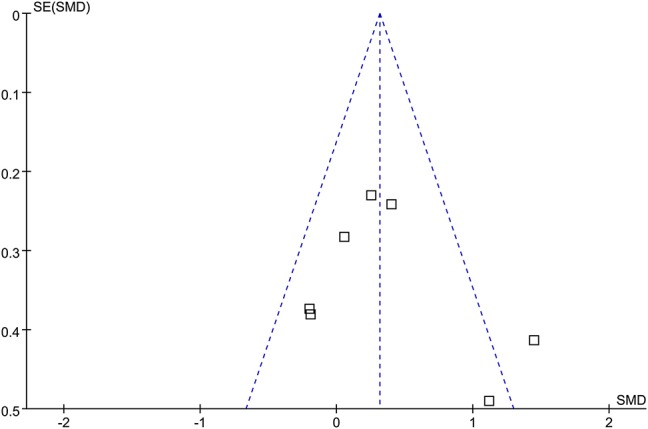
Funnel plot for the meta-analysis of the effect of testosterone supplementation on exercise tolerance as evaluated by the shuttle walk test (SWT) or the six-minute walk test (6MWT).

Forest plot for the meta-analysis of supplementation on parameters of exercise capacity was shown in Figure 7. The plot seems symmetry on visual inspection, suggesting low risk of publication bias. Potential publication biases of meta-analyses of the other outcomes were difficult to estimate because limited numbers of RCTs (3-6) were included.

## Discussion

In this updated meta-analysis of RCTs, we found that testosterone supplementation was not associated with significantly improved exercise tolerance in CHF patients, as evidenced by the increment of 6MWT and SWT. Results of sensitivity analyses limited to studies of male CHF patients showed similar results. In addition, subgroup analyses demonstrated that testosterone supplementation did not significantly affect the exercise capacity in male CHF patients with baseline TT ≥ 10 nmol/L or those with baseline TT <10 nmol/L. However, subgroup analyses according to the TT level at endpoint showed that male patients with endpoint TT ≥ 25 nmol/L was associated with improved exercise capacity, but not for those with endpoint TT <25 nmol/L. Moreover, supplementation of testosterone also did not significantly improve NYHA functional classification or MLHF Score, indicating an improved quality of life. In addition, testosterone supplementation in CHF was not associated with improved cardiac function since no significant changes of LVEF or BNP were noticed in treated patients. Also, testosterone supplementation did not significantly affect the composite outcome of death or HF rehospitalization. Taken together, these results suggested that testosterone supplementation within physiological range was not associated with improved exercise capacity, quality of life, cardiac function, or clinical outcome in CHF patients.

Results of our study did not show that testosterone supplementation was associated with significantly improved exercise tolerance in CHF patients as evaluated by walking test, which is not consistent with the results of the previous meta-analysis (22). Results of meta-analysis only suggest a trend of improved exercise capacity in CHF patients after testosterone supplementation as compared to controls (p = 0.07). Consistently, we also did not find that testosterone supplementation is associated with a significantly improved cardiac function. These results indicated that the potential benefit of testosterone supplementation in CHF patient, if any, is probably only a moderate efficacy on exercise capacity. Moreover, this possible benefit may be related to the influence of testosterone supplementation on peripheral tissues instead of a direct effect on myocardium. Interestingly, results of previous animal studies demonstrated that testosterone administration may dose-dependently prevent the loss of bone and skeletal muscles (31, 32), particularly in those with androgen deficiency. In addition, an early meta-analysis including 29 RCTs of middle-aged men showed that compared to placebo, supplementation of testosterone is associated with a tendency of improvement in muscle strengths at the leg/knee extension and handgrip of the dominant arm, accompanied with improved bone mineral density at the lumbar spine (33). On the other hand, abnormalities of skeletal muscle volume and endurance have been correlated with the impairment of exercise capacity in CHF patients (34). Besides, results of our study indicated that testosterone supplementation in CHF may be associated with increased SBP. In view of the fact that lower SBP has been established as a predictor of poor prognosis in CHF (35), increasing SBP may possibly be a potential benefit of testosterone supplementation in CHF patient. Again, since no significant improvement of cardiac function were observed after testosterone supplementation, we considered the increment of SBP after supplementation of testosterone may be also depended on its efficacy on volume and strength of peripheral skeletal muscle. This is not surprising since the contraction of peripheral skeletal muscle may serve as a secondary pump to maintain the BP, particularly in the elderly (36, 37). Furthermore, results our meta-analysis did not support that testosterone supplementation in CHF patients was associated with significantly improved prognosis. This is not surprising since testosterone supplementation was not associated with any improvement in the exercise capacity, cardiac function, or quality of life in CHF patients as evidenced by the results of our meta-analysis. However, only eight RCTs with 332 CHF patients were included with a mean follow-up duration of 6.2 months, which may be underpowered to detect a significant influence of testosterone supplementation on clinical outcomes. Further large-scale RCTs with long follow-up durations are needed to validate the findings of the meta-analysis.

Aiming to evaluate whether the baseline gonad status of male CHF patients may affect the influence of testosterone supplementation on the exercise capacity, we performed subgroup analysis according to the baseline serum TT levels of the treated patients. Results showed that testosterone supplementation did not improve exercise capacity in male patients with baseline TT ≥ or < 10 nmol/L, which may reflect that the efficacy of testosterone supplementation on exercise capacity may not be affect by the baseline level of TT. Subsequently, we analyzed whether the efficacy of testosterone could be affected by TT at endpoint. Results of subgroup analysis showed that male patients with endpoint TT ≥ 25 nmol/L was associated with improved exercise capacity, but not for those with endpoint TT < 25 nmol/L. Since only three studies were available for the subgroup analysis, the results should be interpreted very cautiously. Nevertheless, these findings may demonstrate that supplementation of testosterone over the physiological range of testosterone could improve exercise capacity in male CHF patients, but not for studies with testosterone supplementation within a physiological range. However, since high-dose or long-term supplementation of testosterone has been suspected to expose the patients to higher risk of adverse cardiovascular diseases (38) or progression of prostate cancer (39). The benefit and risk of testosterone supplementation over the physiological range in male CHF should be cautiously determined in future studies. From this point of view, the finding for the improved exercise capacity after testosterone supplementation in the previous meta-analysis by Toma et al. (22) was mainly driven by the study with female patients (17) and the study with testosterone supplementation over the physiological range (14). Accordingly, it could be estimated that subsequent RCTs included male patients and the testosterone supplementation was likely to be within the physiological range (18, 19, 21), which therefore was not likely to show a significantly improved exercise capacity in these studies. By including these studies in this updated meta-analysis, the overall efficacy of testosterone supplementation on exercise capacity was shown to be non-significant.

Our study has limitations which should be considered when interpreting the results. Moreover, carefully consideration of these limitations may provide some implication for the future RCTs regarding the same topic. Firstly, although our meta-analysis updated previous one by including four more newly published RCTs, the number of the included RCTs and the sample sizes of participants in each RCT remain limited, which prevented us from conducting further analyses to explore the source of heterogeneity among the included RCTs. Secondly, significant heterogeneity existed among the included RCTs when pooling the results of exercise tolerance. Variations of clinical characteristics of the included RCTs may contribute to the heterogeneity, such as the circulating testosterone status of the participants at baseline, concurrent medications, administrative routes and doses of testosterone supplementation, as well as the treatment durations. Of these, baseline circulating testosterone level seems to particularly important. Because testosterone deficiency seems to be an important prognostic factor in CHF patients, theoretically, testosterone may only be effective in patients with testosterone deficiency. However, no consensus has been reached for the definition of testosterone deficiency in CHF patients (40, 41). Therefore, efforts are needed to evaluate the potential efficacy of testosterone supplementation in CHF patients with androgen deficiency. Moreover, the follow-up duration of the included RCTs are relatively short (mostly within 3 to 6 months). Long-term efficacy of testosterone supplementation in patients with CHF, particularly on clinical outcome, deserves further investigation. Finally, although no significant adverse events that related to testosterone supplementation were reported in any of the included RCTs, the cardiovascular safety of testosterone supplementation remains to be evaluated in long-term studies (9).

In conclusion, results of the meta-analysis indicated that testosterone supplementation within a physiological range is not associated with significantly improved exercise capacity, cardiac function, quality of life, or clinical outcome in CHF patients. Results of our study should be validated in large-scale RCTs including male CHF patients of androgen deficiency.

## Data Availability Statement

All datasets generated for this study are included in the article/supplementary material.

## Author Contributions

JT and WB conceived and designed the study. JT and XL performed database search, literature identification, data extraction, and statistical analyses. JT drafted the manuscript. XL and WB critically revised the manuscript. All authors analyzed and interpreted the results and approved the manuscript for submission.

### Conflict of Interest

The authors declare that the research was conducted in the absence of any commercial or financial relationships that could be construed as a potential conflict of interest.
